# Application of and Clinical Research on Enhanced Recovery After Surgery in Perioperative Care of Patients With Supratentorial Tumors

**DOI:** 10.3389/fonc.2021.697699

**Published:** 2021-06-28

**Authors:** Jingmi Wu, Weina Zhang, Jie Chen, Hui Fei, Hong Zhu, Haofen Xie

**Affiliations:** ^1^ Department of Neurology, Ningbo First Hospital, Ningbo, China; ^2^ Department of Theater, Ningbo First Hospital, Ningbo, China; ^3^ Department of Nursing, Ningbo First Hospital, Ningbo, China

**Keywords:** supratentorial tumors, perioperative care, enhanced recovery after surgery, surgical treatment, safe and effective

## Abstract

**Purpose:**

This study intends to explore the safety and effectiveness of the concept of enhanced recovery after surgery (ERAS) in the perioperative care of patients with supratentorial tumors.

**Methods:**

A total of 151 supratentorial tumor patients were enrolled in this study, and they were divided into control group (n = 75) and observation group (n = 76) according to the random number table method. Patients in the control group received routine neurosurgery care, and patients in the observation group received enhanced recovery after surgery care. The incidence of perioperative complications, postoperative hospital stays, early postoperative eating time, catheter removal time, and time to get out of bed were observed for the two groups of patients, and the quality of postoperative recovery was evaluated.

**Results:**

There was no statistically significant difference in the basic data of the two groups of patients, such as age, gender, lesion location, and condition (P>0.05), and they were comparable. The observation group’s postoperative eating time, catheter removal time, and time to get out of bed were significantly earlier than those of the control group. Postoperative hospital stays and hospitalization expenses were less than those of the control group. There was a statistically significant difference in postoperative hospital stay between the two groups (P<0.05).

**Conclusion:**

Applying the ERAS concept to implement perioperative care for patients with supratentorial tumors is safe and effective. It can not only reduce after-surgical stress and accelerate postoperative recovery, but also shorten hospital stays and reduce hospital costs. It is worthy of clinical application.

## Introduction

Supratentorial tumors account for 80% of neurosurgery operations, and headaches, increased intracranial pressure, epilepsy, and local brain dysfunction are common. The nature of the tumor includes astrocytoma, glioblastoma, meningioma, metastatic tumor, oligodendroglioma, and primary central nervous system lymphoma. Surgical resection is the most important, direct, and effective method for supratentorial tumors.

Enhanced recovery after surgery (ERAS), also known as “rapid recovery after surgery,” (1–3) was first proposed by Danish surgeon Professor Kehlet (4) in 1997. It adopts a series of perioperative optimization treatment measures with evidence-based medical evidence to reduce the physical and psychological trauma of surgical patients, reduce complications, and achieve the purpose of enhanced recovery. A large number of randomized clinical trials and Meta-analysis at home and abroad have confirmed the safety of ERAS and its many advantages in the recovery process of patients (5). ERAS has been introduced to neurosurgery recently. Elayat et al. found that ERAS could reduce the proportion of patients requiring intensive care unit (ICU) stay > 48 hours after supratentorial neurosurgery (6). Hughes et al. demonstrated that an ERAS protocol after elective endoscopic pituitary surgery could reduce length of stay and was associated with high patient satisfaction (7). However, the consensus has not been reached for evidence-based recommendations of it (8).

Under the guidance of the ERAS concept, we reported the safety and effectiveness of enhanced recovery after surgery in the perioperative care of neurosurgery patients with supratentorial tumors to provide the theoretical basis for its application and evaluate its potential value for clinical treatment.

## Materials and Methods

### Research Object

In this study, the patients with supratentorial tumors admitted to the Department of Neurosurgery of our hospital from June 2018 to August 2019 were selected as the main research objects. The included patients were randomly sampled by the “envelope method” according to the random number assigned when they were admitted to the hospital and entered the control group (n = 75) and the observation group (n = 76), respectively. This study complies with the “Declaration of Helsinki of the World Medical Association” and has been approved by the ethics committee of our hospital. All patients signed an informed consent form.

### Enrollment *C*riteria

#### Inclusion Criteria

- Patients with a clear diagnosis of supratentorial tumor, of both genders
- Diagnosed by brain CT and MRI
- Requiring surgical operation.
- Patients of the American Society of Anesthesiologists’ (ASA) physical status I or II
- Older than 18 years
- Able to cooperate, particularly the ability to sign the informed consent voluntarily

#### Exclusion Criteria

- Refusal to participate or withdrawal halfway
- Poor compliance
- Encountering other stress events that may affect the results of the study
- Patients with incomplete case data

### Interventions

#### The ERAS Group

The patients in observation group will be treated under the guidance of the ERAS concept. The details were as follows:

##### ERAS Pathway for Preoperative Care

(1) Preoperative assessment and ERAS education: The nurses completed the nutritional risk screening of the patient by using the visual analog scale and the facial expression scale to evaluate the pain, the Barthel index score, and the DVT risk assessment. The doctors should inform patients of the concept of ERAS and explain the operation methods and complications through PPT, so that the patients could understand the disease and the whole treatment process.
(2) Preoperative airway preparation: The patients should quit smoking 4 weeks before surgery. Training methods such as climbing stairs and blowing balloons were encouraged to increase vital capacity. The patients were taught how to breathe deeply and cough effectively. At the same time, their family members should be known how to assist them to turn over and tap the back effectively.
(3) Prevention of deep vein thrombosis: For patients older than 60 years or admitted to the hospital with a high-risk VTE score, the responsible nurse informs the VTE risk and preventive measures and teaches family members and patients to perform passive exercise training, ankle pump exercises, prepare elastic stockings, and guide correct wearing methods before surgery.
(4) Preoperative gastrointestinal management: Fasting for 6 h before surgery, patients without a history of diabetes orally took 400 ml of carbohydrate solution 3 h before surgery. In addition, cathartic should be used one day before operation and glycerine enema should be used if necessary.
(5) Preoperative medication: The nurses were responsible for asking the patient about the history of allergies and performing a skin test before the surgery. The antibacterial drugs were prepared for intravenous prophylactic application on the day of the operation.
(6) Skin preparation: Firstly, the shampoo containing “4% chlorhexidine” was used to clean the head 3 days before surgery. And then, on the surgery day, the marginal hair was trimmed only. The 3M surgical skin preparation kit was used for partial haircuts (2–3 cm wide along the incision). Next, the rest of the hair was sterilized and the long hair was given a split braid.

##### ERAS Pathway for Postoperative Care

(1) Postoperative monitoring: The NICU care was performed on the first day after surgery. Simultaneously, the vital signs and complications were observed closely. 4 h after awake extubation, the patients should be orally or nasally fed clear liquid nutritional supplements, 30 to 50 ml/time, once every 1 to 2 h. If CT showed no hemorrhage, ischemia, edema, and other intracranial abnormalities 24 h after the operation, the patient could be sent back to the neurosurgery ward.
(2) Postoperative gastrointestinal management: Antiemetics were used routinely 3 days after operation for maintaining the drug concentration in the body and reducing the risk of complications. In order to provide early nutritional support, 100 to 200 ml/time should be added every 2 to 3 h from 24 h after the operation, and it should be gradually transferred to semi liquid according to the tolerance. For those who have no eating difficulties, ordinary food would be given 72 h after the operation.
(3) Prevention of postoperative deep vein thrombosis: High risk VTE patients should stop using hemostatic drugs 2 days after operation, and use graded pressure socks and intermittent pneumatic pump until discharge. Anticoagulants were routinely used only when patients had obvious thromboembolic symptoms.
(4) Postoperative tube care: The negative pressure drainage ball was removed within 48 h. 12 h after surgery, the patients should be introduced to start bladder function training. The patients should urinate every 3 to 4 h within 24 h after surgery. Furthermore, the central venous catheter was removed within 72 h after surgery.
(5) Postoperative pain management: visual analog scale (VAS) and facial expression scale were used to score postoperative pain and were reevaluated according to the doctor’s analgesic instructions.
(6) Early postoperative getting out of bed activities: The nurses cooperate with the doctors to complete the PQRS score. From leaving the anesthesia and resuscitation room to the patient’s discharge, it is calculated in days to observe whether the patient can take care of himself and perform the Post-operative Quality Recovery Scale (PQRS) Score and archive medical history cards. On the second day after the operation, they can get out of bed, sit quietly and walk by the bed. Patients should be educated to exercise abdominal breathing and urged to turn over and expectorate.

#### The Control Group

The patients undergoing supratentorial tumors in the control group received conventional care according to our center. There was no standard protocol for preoperative care and postoperative care, including skin preparation, intraoperative medications, as well as tube care, pain management, gastrointestinal management, and so on.

### Main Observation Indicators

The responsible nurse records the perioperative complication rate, postoperative hospital stays, early postoperative eating time, catheter removal time, and time to get out of bed. As to patient satisfaction and nurses’ self-efficacy, four PQRS scales are completed by telephone follow-up at the end of the operation when patients initially wake up, on the 3rd day after the operation, 1 week after the operation, and 1-3 months after the operation.

### Statistical Methods

In this study, SPSS 19.0 statistical software was used for data processing. Normally distributed measurement data were expressed as mean ± standard deviation (`x ± s), non-normally distributed measurement data were represented by median M and quartiles (P25, P75), and count data were represented by percentage (%). The t-test was used for comparison between the two groups; the nonparametric test was used for the comparison between groups that did not obey the normal distribution. Chi-square test was used for counting data. P<0.05 indicates that the difference is statistically significant.

## Results

### General Information

A total of 151 supratentorial tumor patients were enrolled in this study, and they were divided into control group (n=76) and ERAS group (n=75) according to the random number table method.

There was no statistically significant difference in the basic data of the two groups of patients, such as age, gender, lesion location, and condition (P>0.05), and they were comparable. Furthermore, in control group, 40 patients had meningiomas, 31 patients had gliomas, and five patients had other tumors. In ERAS group, 38 patients had meningiomas, 30 patients had gliomas, and seven patients had other tumors. The difference of histotypes between the two groups was not significant (p=0.821).

### Comparison of Nursing Outcome Indicators Between the Two Groups

The aim of this study was to assess the indicators including eating time, catheter removal time, time to get out of bed, postoperative hospital stay, and postoperative hospitalization expenses. The assessments were performed on all patients of the two groups. Compared to the control group, the eating time, catheter removal time, and time to get out of bed of ERAS group was significantly earlier.

In **Table 1**, the postoperative hospital stay in the ERAS group was not significantly shortened (P>0.05), while the total hospitalization expenses of patients in the ERAS group were significantly lower than those in the control group (P<0.05). Meanwhile, the patients from the ERAS group spent less in hospitalization, and their expenditure was six times lower than that in the control group as shown in Table 1. Additionally, there was less readmission and reoperation in the ERAS group, but the difference was not significant (p = 0.765 in readmission and p=1.000 in reoperation).

**Table 1:** Clinical outcomes.

	ERAS group(n=75)	Control group(n=76)	P value
LOS (days), median	8	11	<0.001
Total hospital costs (¥), median	148,500	24,688	<0.001
Readmission, n (%)	5(6,7)	7(9,2)	0.765
Reoperation, n (%)	1(1,3)	2(2,6)	1.000

### Perioperative *C*omplications of the Two Groups of *P*atients

The characteristics of the surgical complications were similar in the ERAS group and control group (**Table 2**). All patients received timely symptomatic treatment for complications. In the present study, there was no related death. There was no significant difference between the two groups of patients with complications such as intracranial infection, lung infection, urinary tract infection, and electrolyte disorders (P>0.05). Although the difference was not significant on the rest of the complications, the incidence had a lower tendency after the ERAS treatment.

**Table 2:** Postoperative complications in detail.

	ERAS group (n=75)	Control group (n=76)	P value
Pancreatic fistula, n (%)			0.022
None	28 (37.3)	18 (23.7)	
A	46 (61.3)	48 (63.2)	
B	1 (1.3)	8 (10.5)	
C	0 (0)		
Delayed gastric emptying, n (%)			0.015
None	68 (90.7)	55 (72.4)	
A	2 (2.7)	12 (15.8)	
B	1 (1.3)	4 (5.3)	
C	4 (5.3)	5 (6.6)	
Chylous leakage, n (%)	2 (2.7)	12 (15.8)	0.681
Intestinal fistula, n (%)	0 (0)	2 (2.6)	0.5000
Abdominal abscess, n (%)	4 (5.3)	8 (10.5)	0.368
Digestive abscess, n (%)	1 (1.3)	2 (2.6)	1.000
Incision infection, n (%)	6 (8.0)	10 (13.2)	0.429
Venous thrombosis, n (%)	0 (0)	2 (2.6)	0.500
Pneumonia, n (%)	4 (5.3)	13 (17.1)	0.037
Fever, n (%)	5 (6.7)	8 (10.5)	0.398
Mortality	0	0	

## Discussion

### ERAS Could Relieve Patients’ Anxiety Before Surgery

Most patients will experience varying degrees of panic and anxiety due to ignorance of the operation and worry about the safety of the operation. Effective communication before surgery is particularly important. Both groups of patients received preoperative education, while the observation group established a neurosurgery ERAS multidisciplinary collaboration group. Under the medical and nursing collaborative management, the publicity and education were significantly better than the control group, and the postoperative cooperation ability and self-recovery enthusiasm were high. The responsible nurse made a patient supervision card and participated in the postoperative supervision throughout the whole process, and the sense of self-efficacy was higher than that of the control group.

Additionally, with the increasing demand of patients for anesthesia knowledge before surgery, it is increasingly important for medical staff to master anesthesia knowledge. Studies find that 75% of patients want to obtain comprehensive knowledge of anesthesia, and 80% of patients want to strengthen the flexible preoperative health education with a nurse’s oral explanation of anesthesia knowledge. Some scholars (9) believe that patients hope to obtain many aspects of knowledge about anesthesia during preoperative visits. Among them, the systematic and detailed method of issuing health manuals is most favored by patients. Therefore, pre-operative anesthesia education for the patient also helps to promote the patient’s recovery. Under the guidance of the ERAS, medical staff mastering relevant anesthesia knowledge and providing psychological care for patients before surgery can help patients relieve anxiety and minimize the psychological barriers caused by surgery (10).

### ERAS Could Reduce the Occurrence of Complications After Surgery

Prolonged fasting puts patients in a metabolic stress state, which can cause insulin resistance, which is not conducive to reducing the incidence of postoperative complications (11). Some studies believe that oral intake of an appropriate amount of clear liquid nutritional supplements 4 h after surgery not only does not increase patient discomfort but also promotes rapid recovery of gastrointestinal function (12), reduces hunger, and improves comfort. In the control group, two patients with hypoglycemia caused by fasting before operation were treated with intravenous infusion, which increased the chance of infection, while the observation group did not have hypoglycemia.

Pain is one of the main stress factors for patients after surgery, which can lead to early postoperative getting out of bed or delay in discharge time, hindering the recovery of surgical patients after surgery and affecting the quality of life of patients after surgery. Therefore, pain treatment is a very important part of ERAS (13). The nurse assessed the pain twice a day (once in the morning and once in the evening) and the presence or absence of adverse reactions to analgesic drugs for 1 to 3 days after the operation. The doctor reviewed and adjusted the analgesic drugs in time based on the scoring results. Good analgesia promotes early activities of patients, reduces complications, improves self-care ability in daily life, and accelerates recovery.

Early getting out of bed helps improve blood circulation throughout the body, can effectively reduce the occurrence of complications, such as deep vein thrombosis and pulmonary infection, promote patient recovery, shorten postoperative hospital stay, improve quality of life, and reduce economic pressure (14–16). Respiratory system management is an important part of ERAS and runs through the entire operation period. Research results show that 37.8% of surgical patients have pulmonary complications (17). Active intervention for high-risk patients can help improve lung function and tolerance to surgery, significantly reduce postoperative pulmonary complications, and shorten hospital stay. Malignant tumors, complicated surgery, chemotherapy, and prolonged bed rest are risk factors for venous thromboembolism (18, 19). If patients with risk factors do not have preventive antithrombotic therapy, the incidence of postoperative deep vein thrombosis can reach 30% and the incidence of fatal pulmonary embolism is nearly 1%. It is recommended that middle- and high-risk patients (Caprini score t>3 points) start preventive antithrombotic therapy 2 to 12 h before surgery and continue medication until discharge from the hospital or 14 days after surgery (20, 21). In this study, the complications were compared between the two groups. The observation group had no pulmonary infection or deep vein thrombosis. The results proved to be better than the control group.

### The Dilemma of ERAS in Clinical Application

By consulting domestic and foreign literature (22, 23), it is found that the application of ERAS in neurosurgery is rarely reported nationwide or even worldwide. Li Chaoyue et al. (24) believe that neurosurgery patients are seriously ill and have long treatment cycles, which are the main reasons why ERAS has not attracted enough attention and clinical application. The research of Jiang Zhiwei and Li Jieshou (25) points out that people’s adherence to traditional ideas has become a serious obstacle to the promotion of ERAS, and the knowledge, attitudes, and behaviors of medical staff toward ERAS have become important factors affecting the application and effectiveness of ERAS (26). Multidisciplinary cooperation and medical-nursing collaborative management play an important role in ensuring the quality of ERAS. Departments should promote the training of medical and nursing staff in ERAS knowledge and strengthen medical-nursing exchanges.

## Conclusions

Applying the ERAS concept to implement perioperative care for patients with supratentorial tumors is safe and effective. It can not only reduce after-surgical stress and accelerate postoperative recovery but also shorten hospital stays and reduce hospital costs. It is worthy of clinical application.

## Limitations

This research still has the following shortcomings. First of all, this study is a single-center clinical study with a small sample size, and it is still necessary to increase the sample size and conduct multi-center clinical research. Second, the clinical follow-up time of this study is relatively short. Finally, the patients in control group did not follow a special protocol of conventional care which would born lots of bias. Thus, further clinical studies with larger sample size are really needed.

## Data Availability Statement

The original contributions presented in the study are included in the article/supplementary material. Further inquiries can be directed to the corresponding author.

## Ethics Statement

The studies involving human participants were reviewed and approved by Ningbo First Hospital. The patients/participants provided their written informed consent to participate in this study.

## Author Contributions

JW has made substantial contributions to conception and design. WZ and JC participated in the acquisition of data and analysis and interpretation of data. JW, HF, and HZ have been involved in drafting the manuscript and revising it critically for important intellectual content. HF has given final approval of the version to be published. All authors contributed to the article and approved the submitted version.

## Funding

This work was supported by Ningbo Medical Science and Technology Project “Construction and Evaluation of Community Integrated Service Model for Day Surgery” [grant numbers 2017T07]. This work was supported by Zhejiang Medical Science and Technology Project “Construction and Application of Day-Surgery Continuing Nursing Information System under the Hospital and Community Integration Model” (grant 2019KY566). This work was supported by Nursing Discipline Research Project of the Journal of the Chinese Medical Association “Application Research of Multidisciplinary Accelerated Rehabilitation Nursing Model in Patients with Gastrointestinal Cancer” (grant CMAPH-NRI2019049).

## Conflict of Interest

The authors declare that the research was conducted in the absence of any commercial or financial relationships that could be construed as a potential conflict of interest.

## Abbreviations

ERAS, enhanced recovery after surgery; VAS, visual analog scale; PQRS, Post-operative Quality Recovery Scale.
