# Corrigendum: Application of and Clinical Research on Enhanced Recovery After Surgery in Perioperative Care of Patients With Supratentorial Tumors

**DOI:** 10.3389/fonc.2022.863458

**Published:** 2022-04-28

**Authors:** Jingmi Wu, Weina Zhang, Jie Chen, Hui Fei, Hong Zhu, Haofen Xie

**Affiliations:** ^1^ Department of Neurology, Ningbo First Hospital, Ningbo, China; ^2^ Department of Theater, Ningbo First Hospital, Ningbo, China; ^3^ Department of Nursing, Ningbo First Hospital, Ningbo, China

**Keywords:** supratentorial tumors, perioperative care, enhanced recovery after surgery, surgical treatment, safe and effective

In the original article, there was a mistake in **Tables 1** and **2** as published. There were errors with the data calculation in both tables**.** The corrected **Tables 1** and **2** appear below.

**Table 1:** Clinical outcomes.

	ERAS group (n=75)	Control group (n=76)	P value
Postoperative Hospital Stay (d)	12.51 ± 3.52	13.43 ± 3.31	0.09739
Total Hospital Costs (¥)	51128.43 ± 14193.91	57916.16 ± 14259.5	0.003907
Postoperative Eating Time (d)	1.11 ± 1.53	1.53 ± 0.69	0.0001024
Removal Time of Postoperative Urinary Catheter (d)	2.22 ± 2.57	3.53 ± 2.60	0.002282
Time of Getting Out of Bed after Operative (d)	2.56 ± 2.66	4.13 ± 3.42	0.001993

**Table 2:** Postoperative complications in detail.

	ERAS group (n=75)	Control group (n=76)	P Value
Intracranial Infection	2	3	1
Lung Infection	0	5	0.0584026
Urinary Tract Infection	1	2	1
Electrolyte Disorders	1	3	0.61998311
Intramuscular Vein Thrombosis of Lower Extremity	0	1	1
Pleural Effusion	2	2	1
Hypoglycemia	0	2	0.49668874

In the original article, there was an error. Due to the change of table data, the description of the data needs to be corrected.

A correction has been made to **Results, Comparison of Nursing Outcome Indicators Between the Two Groups**, Paragraph 2. The corrected paragraph appears below.

“In **Table 1**, the postoperative hospital stay in the ERAS group was not significantly shortened (P>0.05), while the total hospitalization expenses of patients in the ERAS group were significantly lower than those in the control group (P<0.05). Meanwhile, the patients from the ERAS group spent less in hospitalization, and their expenditure was six times lower than that in the control group as shown in **Table 1**. Additionally, there was less readmission and reoperation in the ERAS group, but the difference was not significant (p = 0.765 in readmission and p=1.000 in reoperation)”.

A correction has also been made to **Results, Perioperative Complications of the Two Groups of Patients**, Paragraph 1. The corrected paragraph appears below.

“The characteristics of the surgical complications were similar in the ERAS group and control group (**Table 2**). All patients received timely symptomatic treatment for complications. In the present study, there was no related death. There was no significant difference between the two groups of patients with complications such as intracranial infection, lung infection, urinary tract infection, and electrolyte disorders (P>0.05). Although the difference was not significant on the rest of the complications, the incidence had a lower tendency after the ERAS treatment”.

The authors apologize for this error and state that this does not change the scientific conclusions of the article in any way. The original article has been updated.

## Publisher’s Note

All claims expressed in this article are solely those of the authors and do not necessarily represent those of their affiliated organizations, or those of the publisher, the editors and the reviewers. Any product that may be evaluated in this article, or claim that may be made by its manufacturer, is not guaranteed or endorsed by the publisher.

